# Meeting of the Medical Association of Georgia

**Published:** 1905-04

**Authors:** 


### MEETING OF THE MEDICAL ASSOCIATION OF GEORGIA.

THE Medical Association of Georgia will hold its annual session at Atlanta on April 19th, 20th and 21st. It will probably be one of the most, if not the most, important meetings in the history of the Association. The adoption or rejection of the proposed new constitution will be the chief concern of the meeting, and some final decision in the matter is confidently expected.

The program has been arranged to admit of ample time for the disputants to present their arguments and also for general discussion. The literary features of the program indicate a preponderance of papers from the Atlanta contingent and it is to be hoped that the prompting of hospitality and good taste will cause it to yield in the matter of favored positions to those who have come from a distance to read their papers.

The local committee of arrangement, consisting of George H. Noble, chairman ; E. B. Block, W. S. Goldsmith, S. A. Visanska and Bernard Wolff, have been occupied in arranging for the entertainment of the visiting members and can assure them a pleasant time. The members of the association resident in Atlanta will one and all unite in expressing a most cordial welcome, and will exert themselves to contribute to the comfort and pleasure of the visiting members.

---

We publish elsewhere a letter from Dr. George H. Simmons, General Secretary of the A. M. A., and also one from Dr. J. N. McCormack, chairman of the committee on reorganization of the A. M. A. The letters are written in reply to a request from Dr. Louis H. Jones for an official definition of the attitude and relation of the American Medical Association to the Medical Association of Georgia.

These letters will be read with interest, as they proceed from authoritative sources and deal with an aspect of the reorganization plan that many of those interested are keenly desirous of having explained.

---

The Anti-Tuberculosis League of America will meet under the presidency of Dr. George Brown, in Atlanta, April 18. Elaborate preparations are being made for the entertainment of guests and no effort will be spared to make their stay pleasant and profitable. Banks and newspapers and other leading institutions of the city are contributing liberally, and there is every prospect of an enthusiastic gathering, with results in the matter of disseminating knowledge among the laity on the subject of tuberculosis that will greatly aid the campaign against it.

---

Dr. Frank H. Sims, a well-known physician of Atlanta, died of pneumonia February 5th. Dr. Sims was a native of Mobile,

Ala., and was forty-three years of age at the time of his death. He received his medical education at the Southern Medical College, and subsequently in Europe. He adopted the specialty of eye, ear, nose and throat, and practiced it in Atlanta for twelve years. He enjoyed the affection and esteem of his fellow practitioners, and his untimely death is deplored.

